# Ongoing Increase in Incidence of Diabetes in Austrian Children and Adolescents (1989–2021): Results from a Nationwide Registry

**DOI:** 10.1155/2023/4616903

**Published:** 2023-08-18

**Authors:** Katrin Nagl, Thomas Waldhör, Sabine E. Hofer, Nicole Blauensteiner, Maria Fritsch, Elke Fröhlich-Reiterer, Birgit Rami-Merhar

**Affiliations:** ^1^Department of Pediatric and Adolescent Medicine, Medical University of Vienna, Vienna, Austria; ^2^Department of Epidemiology, Centre of Public Health, Medical University of Vienna, Vienna, Austria; ^3^Department of Pediatrics 1, Medical University of Innsbruck, Innsbruck, Austria; ^4^Department of Paediatrics and Adolescent Medicine, Medical University of Graz, Graz, Austria

## Abstract

**Introduction:**

Since there is no uniform global diabetes trend in childhood and adolescence, regional epidemiological surveys of diabetes incidences are important. In Austria, the incidences of type 1 diabetes (T1D), type 2 diabetes (T2D), and other forms of diabetes have been recorded for decades.

**Methods:**

To analyze recent developments of diabetes incidence within the decades long-standing Austrian nationwide prospective population-based incidence study for diabetes in children aged <15 years. We estimated time trends of age-standardized rates from 1989 to 2021 for T1D and T2D by joinpoint analysis. Annual percent changes (APCs) were calculated. Case ascertainment was 97%.

**Results:**

We observed an unusual increase of T1D incidence in the year 2021, reaching a peak of 28.7/100,000/PY (person years). From 2011 to 2020, there had been a constant plateau phase in the total cohort (APC 0.78, 95% CI [−0.99, 2.58], *p* = 0.379), which had followed a steep increase of T1D incidence (APC 4.6, 95% CI [3.94, 5.19], *p* < 0.001) from 1989 to 2011. Age-specific differences in T1D incidence development were observed. For the first time, we observed a statistically significant constant increase in T2D during the observation period (APC 3.47, 95% CI [0.76, 6.26], *p* = 0.014). Other forms of diabetes are two times more common than T2D in this age group.

**Conclusion:**

The incidence of T1D in Austrian children <15 years is still increasing and showed a peak in 2021. For the first time, a significant increase in pediatric T2D was observed in Austria.

## 1. Introduction

The Austrian Diabetes Incidence Registry is a nationwide registry that has collected population-based diabetes incidence data for type 1 diabetes (T1D), type 2 diabetes (T2D), and other forms of diabetes for young people aged <15 years over three decades. The last publication of the Austrian Diabetes Incidence Study Group [1] reported annual diabetes incidences up to 2017. Comparable to other countries, a continuous increase in the T1D incidence with a certain leveling off compared to previous years had been observed [2–7].

Regional studies on the incidence of T1D are necessary because there is no uniform global T1D incidence trend in childhood [8]. Knowledge about the T1D pathogenesis and influencing factors may be derived studying differences between regional T1D time trends [9]. However, besides genetic factors, little is known about causes of T1D [10]. The extent to which a severe acute respiratory syndrome coronavirus 2 (SARS-CoV-2) infection could promote the development of diabetes is the subject of current discussions and research [11, 12]. Additionally, other virus strains relevant to children potentially affecting the incidence of diabetes might also have changed their pattern of circulation following measures during the pandemic [13].

Furthermore, a connection between rotavirus infections and T1D incidence, as well as a protective effect of rotavirus vaccinations, has also been discussed in recent years [1, 14–16].

We, therefore, analyzed recent time trends of diabetes incidences while focusing on T1D and T2D among children and adolescents under the age of 15 years using the Austrian prospective population-based incidence registry.

## 2. Methods

Since 1989, all children up to the age of 15 years who are newly diagnosed with diabetes in Austria have been prospectively registered in the Austrian Diabetes Incidence Registry. T2D and other forms of diabetes have been included in the registry since 1999.

As described in previous publications of the Austrian Diabetes Incidence Study Group [1, 17, 18], the register collects the following parameters of patients at the time of diagnosis: date of birth, gender, date of diagnosis, date of first insulin administration, clinical symptoms of diabetic ketoacidosis (hyperventilation, unconsciousness), height, weight, and laboratory values (glucose, pH, HCO_3_, ketones, HbA1c and diabetes-specific antibodies, family history of diabetes).

The completeness of ascertainment over time was determined using the capture–recapture method recommended by the WHO Diamond Project [19]. From 1999 to 2007, this was >93%; from 2008 to 2013, there was a completeness of case assessment of 97% [7].

As previously described in more detail [1], patients were classified according to American Diabetes Association (ADA) criteria [20, 21]. All cases initially classified as unclear (unclassifiable) type, T2D, or other specific types of diabetes were re-evaluated. The reporting physicians were asked to recheck the diagnosis according to the ADA classification criteria and, if necessary, to incorporate findings from clinical follow-up care, the long-term course, the treatment modalities, and additional results (e.g., genetic tests).

So far, the study periods 1989–1999 [22], 1999–2007 [23], and 2008–2017 [1] have been evaluated for time trends of diabetes incidences. The present study considers the entire study period from January 1, 1989 to December 31, 2021. Rotavirus vaccination coverage (%) data were assessed for the time span of 2007–2021 as previously described [1]. Since July 2007, the costs of rotavirus vaccinations for all children aged 7 weeks to 6 months have been covered by public health insurance in Austria. However, individual data on the vaccination status are not available in Austria.

### 2.1. Statistical Analysis

Annual incidences for T1D and T2D were calculated in total and separately for the three age groups: 0–4, 5–9, and 10–15 years.

Time trends of age-standardized rates, as well as annual percent change (APC) and *p*-values, were estimated by joinpoint analysis (Statistical Research and Applications Branch, National Cancer Institute, Joinpoint Regression Program, version 4.7.0.0). Maximum number of joinpoints was set to two, and minimum number to either side of the end and between joinpoints to two, respectively. Denominator values for calculating rates (i.e., number of boys and girls aged 0 to <15 years) were obtained from the National Population Registry (Statistics Austria) [24]. Age-standardized rates were calculated by year using the European standard population [25]. As in previous publications [1, 17], slopes of trends modeled by joinpoint analysis are described by APC and 95% confidence intervals. Subgroup analyses were performed according to sex and age group (0–4, 5–9, and 10–14 years truncated) in patients with T1D. Ninety-five percent confidence intervals were calculated for proportions as well as means. Figures and all other calculations were done in SAS version 9.4 [26]. Rates are given per 100,000 person years (PY).

Rotavirus vaccination coverage percentage was calculated by the sum of the number of vaccinations divided by two or three depending on the type of vaccination and then by the number of newborns in the corresponding year [1]. The significance level was set to 5%. No adjustment for multiple tests was done and, therefore, *p*-values should be interpreted exploratorily only.

## 3. Results

From 1999 to 2021, 5,888 Austrian children aged younger than 15 years were diagnosed with diabetes (see Table 1).

### 3.1. Incidence of Type 1 Diabetes

**Figure 1:**
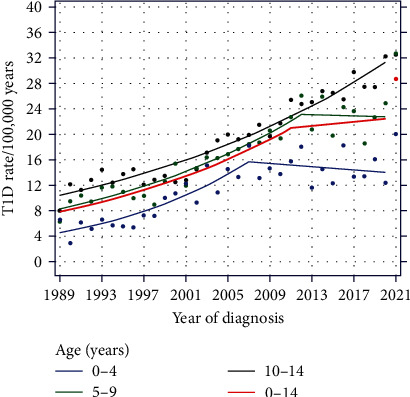
Age-standardized T1D incidence of Austrian children <15 years (1989–2021) by age groups.

In 2021, the Austrian T1D incidence in the total cohort of children <15 years has reached an all-time high of 28.7/100,000 PY. This peak incidence was preceded by a constant plateau phase for the period 2011–2020 (APC 0.78, 95% CI [−0.99, 2.58], *p* = 0.379), which had replaced the steep increase in T1D incidence in the years 1989–2011 (APC 4.56, 95% CI [3.94, 5.19], *p* < 0.001) (Figure 1).

APCs were only calculated up to 2020 due to a sudden increase in incidence in 2021 when compared to previous years. This increase would unduly distort the APC.

Standardized T1D incidence for the whole observation period per age group is shown in *Supplementary 1*. Age groups were divided into the three defined age groups: 0–4, 5–9, and 10–15 years. Age-specific differences in the annual T1D incidence are shown in Figure 1.

### 3.2. Incidence of Type 1 Diabetes: Per Age Group

For all age groups, there was an increase in the incidence of T1D in 2021 compared to previous years. Especially in the age group of 5–9 years, the increase in T1D incidence was extraordinarily steep in 2021 and amounted to 32.7/100,000 PY, corresponding to an increase of 40% compared to previous years. From the years 2012–2020, there had been constant plateau phase with an average annual incidence of 22.97 ± 2.70/100,000 PY (APC −0.21, 95% CI [−3.22, 2.91], *p* = 0.89). Preceding this, from 1989 to 2012, there had been a phase of continuous incidence growth in the years 1989–2012 with an APC of 4.57, 95% CI [3.74, 5.40], (*p* < 0.001).

In contrast, the increase of annual T1D incidence in the group 10–15 years of age had been constant throughout the whole observation period with an APC of 3.62, 95% CI [3.23, 4.01], (*p* < 0.001). With few exceptions (see *Supplementary 1*), this age group had the highest annual incidences among children and adolescents.

Throughout the extended observation period, the dynamics within the age group of 0–4 years diverge from those observed in other age groups. From 1989 to 2007, there had been a steep increase in T1D incidence in the 0–4-year age group (APC 7.10, 95% CI [5.13, 9.11], *p* < 0.001), and there was a slight, nonsignificant decrease from 2007 to 2020 (APC −0.87, 95% CI [−3.18, 1.49], *p* = 0.45), causing a flattening of the curve. Despite the prior decline in incidence, in 2021, the T1D incidence in children aged younger than 4 years was again increasing and reaching a peak of 20.06/100,000 PY.

### 3.3. Type 2 Diabetes

**Figure 2:**
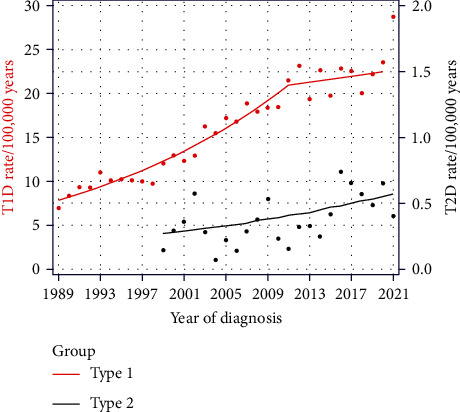
Age-standardized incidence of type 1 and type 2 diabetes in Austrian children aged <15 years (1999–2021).

While 94.3% of all diabetes cases were diagnosed as T1D, only 1.8% were classified as T2D according to ADA criteria. From 1999 to 2021, annual T2D incidences ranged from 0.07 to 0.74/100,000 PY. Despite a quite high variation, there was a constant significant increase in the incidence for T2D with an APC of 3.47, 95% CI [0.75, 8.26], (*p* = 0.014), as shown in Figure 2. Standardized T2D incidence are shown in *Supplementary 2*.

### 3.4. Other Forms of Diabetes

In comparison to T2D, about twice as many cases were classified as other forms of diabetes (3.9%). Most of these cases were diagnosed with maturity onset diabetes of the young (MODY) type 2, MODY3, or cystic fibrosis-related diabetes (CFRD).

## 4. Discussion

### 4.1. Steep Increase of T1D Incidence

The sharp increase of new cases of T1D in 2021 is alarming. Even compared to fluctuations in incidence of pediatric T1D in Austria over the entire observation period, the high numbers for 2021 clearly stand out.

Studies from some other registries are also presenting similar increases in T1D incidence. In Finland, the incidence of children registered to the Finnish Pediatric Diabetes Registry rose from 52.3/100,000 PY during the reference period in 2014–2019 to 61.0/100,000 PY in 2020–2021 [27]. Compared to the forecasted incidence based on the decade before, the actual T1D incidence in Czechia was also well above during the pandemic period 2020/2021 [28, 29]. Similar results were documented in Italy [30], Montenegro [31], and Florida [32, 33]. However, the SWEET registry, including participating centers worldwide, analyzed data from 2018 to 2021 and recently reported that the slope of the T1D incidence remained unchanged during the pandemic, while a change in seasonality occurred [34]. In Germany, data from the longitudinal prescription database were analyzed to detect changes in the prescription incidence for insulin. No cross-correlations between the COVID-19 incidence and T1D incidence were found [33].

In contrast to most other published studies, which are based on shorter time periods, our study encompasses 32 years and allows description of periods of increase, as well as stagnation of the incidence. Based on this long study period, the steep and sudden increase in T1D incidence in 2021 emerges as particularly striking. So, at least, a temporal association with the COVID-19 pandemic is discernible.

### 4.2. T1D Incidence and SARS-CoV-2

The observed increase in T1D incidence may be rooted in an acceleration of the progression of the disease by a SARS-CoV-2 infection [11]. It is also being discussed that SARS-CoV-2 infections might trigger new-onset autoimmunity. In fact, a large, retrospective cohort study showed an increased occurrence of various autoimmune diseases in previously unvaccinated adults within 6 months after a PCR-confirmed SARS-CoV-2 infection [35]. However, in a large, cross-sectionally designed study based on screening children and adolescents in Bavaria and Colorado, no association was found between previous SARS-CoV-2 infections and presence of beta-cell antibodies [33, 36, 37].

Other studies, however, show that a SARS-CoV-2 infection can lead to direct damage to the pancreatic beta cells via angiotensin-converting enzyme (ACE) receptors [38]. SARS-CoV-2 infection might also set a cytokine cascade in motion, which leads to immune-mediated damage [39]. This SARS-CoV-2-associated pancreatic autoimmunity is discussed to be based on the following mechanisms: molecular mimicry, bystander activation, and chronic destruction of beta cells [11].

Some studies have shown a direct connection between a past, recent SARS-CoV-2 infection and a subsequent T1D disease manifestation [12, 40, 41].

### 4.3. Other Possible Causes for the Increase in Incidence

Other possible, but speculative explanations for the steep increase in T1D are indirect effects of the COVID-19 pandemic [42]. Measures taken to combat the spread of SARS-CoV-2 also have led to a change in the circulation of other viruses [13, 43]. Even more, catch-up infections of susceptible children after reopenings of schools, kindergartens, and lifting of lockdown measures between the pandemic waves also may have triggered new islet autoimmunity [29]. Several viruses have been linked to the pathogenesis of T1D. Above all, enteroviruses are suspected to be a precipitating factor in islet autoimmunity [44]. Notably, investigation of the annual distribution of confirmed viral infections between 2017 and 2021 in a German pediatric tertiary center showed a 16-fold increase of rhino/enterovirus infections from 2019 to 2021 [45]. But, also in other parts of the world, relevant enterovirus outbreaks were documented [46]. This increase in rhino/enterovirus infections represented an all-time high and was possibly caused by an increased susceptibility of the pediatric population due to reduced natural exposure during prior lockdown periods [45].

### 4.4. Vaccinations

The stabilization of T1D incidence observed in toddlers from 2007 to 2019 demonstrates a temporal association with the introduction and implementation of rotavirus vaccinations for infants in Austria starting from 2007 [1, 15], see *Supplementary 3*. Starting from 2009, ∼80% of infants residing in Austria received a rotavirus vaccination, on average. A similar trend of stabilization in the group of 5–9-year-olds, beginning around 2011/2012, may also be linked with the introduction of rotavirus vaccinations, albeit with a corresponding time lag. A growing number of studies are dealing with the connection and find either no or a protective effect of rotavirus vaccinations on T1D incidence, as summarized by a recent review on the topic. However, due to the long latency of T1D, scientific proof of a causal relationship is difficult and, therefore, lacking [47].

It remains to be seen to what extent a vaccination against SARS-CoV-2, which is now also approved for very young children, offers protection against the development of long-term sequelae or later autoimmune diseases. In Austria, vaccinations against SARS-CoV-2 for the pediatric population were not available before mid-2021. The approval and availability of COVID-19 vaccines for children and adolescents from the age of 12 years were given in June 2021. A vaccination program for children aged >5 years was started in November 2021. For younger children, COVID-19 vaccines were not available until November 2022.

### 4.5. Increase of T2D Incidence

Another important finding from our current evaluation within the registry is the time trend of T2D incidence, which demonstrates a signficant rise from ∼0.25 cases per 100,000 PY in 1999 to around 0.5 cases per 100,000 PY in 2021. Most likely, the primary reason for this is the increase in obesity in the general population [48]. Recent Austrian data show that up to 7% of preschoolers were overweight, and 3.9% were obese [49].

In the USA, T2D incidence also increased strongly during the COVID-19 pandemic [50]. This pandemic-related effect cannot be read from our data, possibly due to the still very low number of cases. However, triggered by the COVID-19 mitigation measures, there was an increase in obesity in the pediatric sector in Austria [51].

Another potential factor contributing to the rise in T2D incidence in Austria could be the influx of immigrants who may have a higher genetic predisposition to developing T2D [52], as shown in *Supplementary 4* and *Supplementary 5*. Among children under the age of 14 years living in Austria, there has been a strong steady increase in the proportion of those with a migration background since 2007. Around the turn of the millennium, around 10% of the children had migration background; according to data from Statistics Austria, this proportion rose to 20% in 2022 [53].

Regardless, additional efforts must be undertaken to address the emerging “awakening T2D epidemic” among the youth, even if Austria still remains far from the high T2D incidences observed in countries like the USA [54].

There is a pressing need to identify individuals who have a particularly high risk of developing T2D at an early stage. Such individuals could greatly benefit from early therapeutic interventions and preventive programs [54]. There are already successful examples of cost-effective programs in the literature [55, 56]; however, the implementations of such programs are still pending in many regions. The living environment of children should be designed in such a way that healthy nutrition is easy to implement, and that the environment encourages physical activity [56].

### 4.6. Strengths and Limitations

While the strength of our paper clearly lies in the completeness of our data and the exceptionally long observation period, it is essential to acknowledge certain limitations. In lack of any individual COVID-19 data, we have no information whether newly diagnosed children and adolescents with diabetes had previously COVID-19 or other virus infections and, therefore, cannot make any statements regarding causal associations. The same applies to vaccines. Missing information about migration background of the respective patients hinders interpretation of the data. However, data on population level are available, showing a strong and continuous increase in the migration background in the population <15 years [53].

## 5. Conclusion

Despite these limitations, our data provide valuable insights. Epidemiologic observations are essential for the planning of health infrastructure as they help to anticipate trends and act at an early stage. If the current incidence trends persist, there is a likelihood of an upsurge in pediatric cases requiring medical attention, both in terms of T1D and T2D, in Austria.

It is noteworthy that T2D, previously uncommon in Austrian pediatric patients, is now increasingly affecting children and adolescents. Urgent preventive measures, particularly addressing childhood obesity, must, therefore, be taken.

## Figures and Tables

**Table 1:** Newly diagnosed diabetes cases from 1999 to 2021 (<15 years) in Austria.

	Type of diabetes		
	T1D	T2D	Other
*n*	5,550	108	230
%	94.3	1.8	3.9
Female (%) (95% CI)	46.2 (44.9, 47.5)	59.3 (49.4, 68.6)	50.9 (44.2, 57.5)
Mean age (years) (95% CI)	8.5 (8.4, 8.6)	12.7 (12.4, 13.1)	9.4 (8.9, 10.0)

## Data Availability

The data presented in this study are available on request from the corresponding author. The data are not publicly available for data protection reasons.

## Abbreviations

APC: Annual percent change
COVID-19: Coronavirus disease
DKA: Diabetic ketoacidosis
SARS-CoV-2: Severe acute respiratory syndrome coronavirus 2
T1D: Type 1 diabetes
T2D: Type 2 diabetes
PY: Person years.

## Supplementary Materials

Supplementary 1 Cases of newly diagnosed type 1 diabetes per population.

Supplementary 2 Standardized annual incidence rates for type 2 diabetes.

Supplementary 3 Crude T1D incidence 1989–2021 and rotavirus vaccination coverage in Austria.

Supplementary 4 Percentage of inhabitants with non-Austrian nationality below 15 years of age in Austria (graphical description).

Supplementary 5 Percentage of inhabitants with non-Austrian nationality below 15 years of age in Austria (tabular format).
