# Supplementary material for: Ongoing Increase in Incidence of Diabetes in Austrian Children and Adolescents (1989–2021): Results from a Nationwide Registry
Source: Pediatr Diabetes. 2023 Aug 18;2023:4616903. doi: 10.1155/2023/4616903 (PMC12017070; doi:10.1155/2023/4616903)
Supplement: Supplementary 1 — Cases of newly diagnosed type 1 diabetes per population. [file 4616903.f1.docx]

| **Suppl. Table 1: Cases of newly diagnosed Type 1 Diabetes per population and standardized Annual Incidence Rates for Type 1 Diabetes given as 100.000 per person years (PY). divided per age-group** | | | | | | | | | | | | | | | | | | | | | | | | |
| --- | --- | --- | --- | --- | --- | --- | --- | --- | --- | --- | --- | --- | --- | --- | --- | --- | --- | --- | --- | --- | --- | --- | --- | --- |
| Age group | 0-14 years | | | | | | 0-4 years | | | | | | 5-9 years | | | | | | 10-14 years | | | | | |
| Year | N | population | Stand. Rate | Std. Err | 95%CI  LB | 95%CI  UB | N | population | Stand. Rate | Std. Err | 95%CI  LB | 95%CI  UB | N | population | Stand. Rate | Std. Err | 95%CI  LB | 95%CI  UB | N | population | Stand.Rate | Std.Err | 95%CI LB | 95%CI UB |
| 1989 | 93 | 1338323 | 6.97 | 0.72 | 5.56 | 8.38 | 29 | 441900 | 6.56 | 1.22 | 4.17 | 8.95 | 29 | 458770 | 6.32 | 1.17 | 4.03 | 8.61 | 35 | 437653 | 8.00 | 1.35 | 5.35 | 10.65 |
| 1990 | 110 | 1345495 | 8.35 | 0.80 | 6.78 | 9.92 | 13 | 446497 | 2.91 | 0.81 | 1.32 | 4.50 | 44 | 462563 | 9.51 | 1.43 | 6.71 | 12.31 | 53 | 436435 | 12.14 | 1.67 | 8.87 | 15.41 |
| 1991 | 126 | 1362758 | 9.35 | 0.83 | 7.72 | 10.98 | 28 | 455163 | 6.15 | 1.16 | 3.88 | 8.42 | 48 | 462963 | 10.37 | 1.50 | 7.43 | 13.31 | 50 | 444632 | 11.25 | 1.59 | 8.13 | 14.37 |
| 1992 | 127 | 1387111 | 9.30 | 0.83 | 7.67 | 10.93 | 24 | 465341 | 5.16 | 1.05 | 3.10 | 7.22 | 44 | 463092 | 9.50 | 1.43 | 6.70 | 12.30 | 59 | 458678 | 12.86 | 1.67 | 9.59 | 16.13 |
| 1993 | 153 | 1406126 | 11.02 | 0.89 | 9.28 | 12.76 | 31 | 472063 | 6.57 | 1.18 | 4.26 | 8.88 | 54 | 463669 | 11.65 | 1.58 | 8.55 | 14.75 | 68 | 470394 | 14.46 | 1.75 | 11.03 | 17.89 |
| 1994 | 141 | 1414953 | 10.10 | 0.85 | 8.43 | 11.77 | 27 | 473566 | 5.70 | 1.10 | 3.54 | 7.86 | 55 | 464276 | 11.85 | 1.60 | 8.71 | 14.99 | 59 | 477111 | 12.37 | 1.61 | 9.21 | 15.53 |
| 1995 | 143 | 1415837 | 10.23 | 0.86 | 8.54 | 11.92 | 26 | 471248 | 5.52 | 1.08 | 3.40 | 7.64 | 51 | 465545 | 10.95 | 1.53 | 7.95 | 13.95 | 66 | 479044 | 13.78 | 1.70 | 10.45 | 17.11 |
| 1996 | 141 | 1410820 | 10.10 | 0.85 | 8.43 | 11.77 | 25 | 465310 | 5.37 | 1.07 | 3.27 | 7.47 | 47 | 469554 | 10.01 | 1.46 | 7.15 | 12.87 | 69 | 475956 | 14.50 | 1.75 | 11.07 | 17.93 |
| 1997 | 139 | 1400463 | 9.98 | 0.85 | 8.31 | 11.65 | 33 | 455452 | 7.25 | 1.26 | 4.78 | 9.72 | 49 | 474278 | 10.33 | 1.48 | 7.43 | 13.23 | 57 | 470733 | 12.11 | 1.60 | 8.97 | 15.25 |
| 1998 | 135 | 1388452 | 9.76 | 0.84 | 8.11 | 11.41 | 32 | 443270 | 7.22 | 1.28 | 4.71 | 9.73 | 43 | 477277 | 9.01 | 1.37 | 6.32 | 11.70 | 60 | 467905 | 12.82 | 1.66 | 9.57 | 16.07 |
| 1999 | 166 | 1377375 | 12.06 | 0.94 | 10.22 | 13.90 | 43 | 430714 | 9.98 | 1.52 | 7.00 | 12.96 | 60 | 478129 | 12.55 | 1.62 | 9.37 | 15.73 | 63 | 468532 | 13.45 | 1.69 | 10.14 | 16.76 |
| 2000 | 177 | 1365466 | 12.94 | 0.97 | 11.04 | 14.84 | 45 | 419493 | 10.73 | 1.60 | 7.59 | 13.87 | 73 | 475213 | 15.36 | 1.80 | 11.83 | 18.89 | 59 | 470760 | 12.53 | 1.63 | 9.34 | 15.72 |
| 2001 | 167 | 1352356 | 12.35 | 0.96 | 10.47 | 14.23 | 50 | 407842 | 12.26 | 1.73 | 8.87 | 15.65 | 56 | 469088 | 11.94 | 1.60 | 8.80 | 15.08 | 61 | 475426 | 12.83 | 1.64 | 9.62 | 16.04 |
| 2002 | 175 | 1342780 | 12.95 | 0.98 | 11.03 | 14.87 | 37 | 399351 | 9.27 | 1.52 | 6.29 | 12.25 | 68 | 461914 | 14.72 | 1.79 | 11.21 | 18.23 | 70 | 481515 | 14.54 | 1.74 | 11.13 | 17.95 |
| 2003 | 217 | 1333505 | 16.24 | 1.10 | 14.08 | 18.40 | 60 | 396727 | 15.12 | 1.95 | 11.30 | 18.94 | 74 | 451151 | 16.40 | 1.91 | 12.66 | 20.14 | 83 | 485627 | 17.09 | 1.88 | 13.41 | 20.77 |
| 2004 | 208 | 1325997 | 15.55 | 1.08 | 13.43 | 17.67 | 43 | 396819 | 10.84 | 1.65 | 7.61 | 14.07 | 72 | 441022 | 16.33 | 1.92 | 12.57 | 20.09 | 93 | 488156 | 19.05 | 1.98 | 15.17 | 22.93 |
| 2005 | 228 | 1317707 | 17.20 | 1.14 | 14.97 | 19.43 | 58 | 398509 | 14.55 | 1.91 | 10.81 | 18.29 | 73 | 432662 | 16.87 | 1.97 | 13.01 | 20.73 | 97 | 486536 | 19.94 | 2.02 | 15.98 | 23.90 |
| 2006 | 222 | 1312683 | 16.84 | 1.13 | 14.63 | 19.05 | 53 | 399233 | 13.28 | 1.82 | 9.71 | 16.85 | 76 | 428810 | 17.72 | 2.03 | 13.74 | 21.70 | 93 | 484640 | 19.19 | 1.99 | 15.29 | 23.09 |
| 2007 | 245 | 1294718 | 18.89 | 1.21 | 16.52 | 21.26 | 73 | 399500 | 18.27 | 2.14 | 14.08 | 22.46 | 77 | 416818 | 18.47 | 2.11 | 14.33 | 22.61 | 95 | 478400 | 19.86 | 2.04 | 15.86 | 23.86 |
| 2008 | 230 | 1277511 | 17.92 | 1.18 | 15.61 | 20.23 | 52 | 396259 | 13.12 | 1.82 | 9.55 | 16.69 | 77 | 411922 | 18.69 | 2.13 | 14.52 | 22.86 | 101 | 469330 | 21.52 | 2.14 | 17.33 | 25.71 |
| 2009 | 232 | 1261588 | 18.42 | 1.21 | 16.05 | 20.79 | 58 | 396528 | 14.63 | 1.92 | 10.87 | 18.39 | 84 | 407342 | 20.62 | 2.25 | 16.21 | 25.03 | 90 | 457718 | 19.66 | 2.07 | 15.60 | 23.72 |
| 2010 | 230 | 1244870 | 18.46 | 1.22 | 16.07 | 20.85 | 54 | 392853 | 13.75 | 1.87 | 10.08 | 17.42 | 79 | 406779 | 19.42 | 2.19 | 15.13 | 23.71 | 97 | 445238 | 21.79 | 2.21 | 17.46 | 26.12 |
| 2011 | 265 | 1234761 | 21.48 | 1.32 | 18.89 | 24.07 | 62 | 393393 | 15.76 | 2.00 | 11.84 | 19.68 | 92 | 405099 | 22.71 | 2.37 | 18.06 | 27.36 | 111 | 436269 | 25.44 | 2.41 | 20.72 | 30.16 |
| 2012 | 282 | 1224361 | 23.11 | 1.38 | 20.41 | 25.81 | 71 | 393847 | 18.03 | 2.14 | 13.84 | 22.22 | 106 | 406309 | 26.09 | 2.53 | 21.13 | 31.05 | 105 | 424205 | 24.75 | 2.42 | 20.01 | 29.49 |
| 2013 | 235 | 1219363 | 19.39 | 1.27 | 16.90 | 21.88 | 46 | 396879 | 11.59 | 1.71 | 8.24 | 14.94 | 84 | 403639 | 20.81 | 2.27 | 16.36 | 25.26 | 105 | 418845 | 25.07 | 2.45 | 20.27 | 29.87 |
| 2014 | 274 | 1218844 | 22.66 | 1.37 | 19.97 | 25.35 | 58 | 399504 | 14.52 | 1.91 | 10.78 | 18.26 | 105 | 404547 | 25.95 | 2.53 | 20.99 | 30.91 | 111 | 414793 | 26.76 | 2.54 | 21.78 | 31.74 |
| 2015 | 240 | 1226013 | 19.74 | 1.28 | 17.23 | 22.25 | 50 | 406003 | 12.32 | 1.74 | 8.91 | 15.73 | 80 | 403898 | 19.81 | 2.21 | 15.48 | 24.14 | 110 | 416112 | 26.44 | 2.52 | 21.50 | 31.38 |
| 2016 | 283 | 1246847 | 22.83 | 1.36 | 20.16 | 25.50 | 76 | 416123 | 18.26 | 2.10 | 14.14 | 22.38 | 100 | 411101 | 24.32 | 2.43 | 19.56 | 29.08 | 107 | 419623 | 25.50 | 2.47 | 20.66 | 30.34 |
| 2017 | 281 | 1263740 | 22.54 | 1.35 | 19.89 | 25.19 | 57 | 425651 | 13.39 | 1.77 | 9.92 | 16.86 | 98 | 414639 | 23.64 | 2.39 | 18.96 | 28.32 | 126 | 423450 | 29.76 | 2.65 | 24.57 | 34.95 |
| 2018 | 252 | 1273002 | 20.04 | 1.26 | 17.57 | 22.51 | 58 | 432008 | 13.43 | 1.76 | 9.98 | 16.88 | 78 | 418903 | 18.62 | 2.11 | 14.48 | 22.76 | 116 | 422091 | 27.48 | 2.55 | 22.48 | 32.48 |
| 2019 | 281 | 1278692 | 22.22 | 1.33 | 19.61 | 24.83 | 70 | 435133 | 16.09 | 1.92 | 12.33 | 19.85 | 95 | 420286 | 22.60 | 2.32 | 18.05 | 27.15 | 116 | 423273 | 27.41 | 2.54 | 22.43 | 32.39 |
| 2020 | 296 | 1283060 | 23.52 | 1.37 | 20.83 | 26.21 | 54 | 435835 | 12.39 | 1.69 | 9.08 | 15.70 | 106 | 425227 | 24.93 | 2.42 | 20.19 | 29.67 | 136 | 421998 | 32.23 | 2.76 | 26.82 | 37.64 |
| 2021 | 365 | 1285488 | 28.71 | 1.50 | 25.77 | 31.65 | 87 | 433755 | 20.06 | 2.15 | 15.85 | 24.27 | 140 | 428300 | 32.69 | 2.76 | 27.28 | 38.10 | 138 | 423433 | 32.59 | 2.77 | 27.16 | 38.02 |
