# Supplementary material for: Ongoing Increase in Incidence of Diabetes in Austrian Children and Adolescents (1989–2021): Results from a Nationwide Registry
Source: Pediatr Diabetes. 2023 Aug 18;2023:4616903. doi: 10.1155/2023/4616903 (PMC12017070; doi:10.1155/2023/4616903)
Supplement: Supplementary 2 — Standardized annual incidence rates for type 2 diabetes. [file 4616903.f2.docx]

### Suppl. Table 2: Standardized Annual Incidence Rates for Type 2 Diabetes given as 100.000 per person years (PY)

| **Year** | Stand.Rate | Lower CI | **Upper CI** | **Cases** | **Population** |
| --- | --- | --- | --- | --- | --- |
| **1999** | 0.15 | 0.00 | 0.35 | 2 | 1377375 |
| 2000 | 0.29 | 0.01 | 0.58 | 4 | 1365466 |
| 2001 | 0.36 | 0.04 | 0.68 | 5 | 1352356 |
| 2002 | 0.57 | 0.18 | 0.97 | 8 | 1342780 |
| 2003 | 0.28 | 0.01 | 0.56 | 4 | 1333505 |
| 2004 | 0.07 | 0.00 | 0.21 | 1 | 1325997 |
| 2005 | 0.22 | 0.00 | 0.47 | 3 | 1317707 |
| 2006 | 0.14 | 0.00 | 0.34 | 2 | 1312683 |
| 2007 | 0.29 | 0.01 | 0.57 | 4 | 1294718 |
| 2008 | 0.38 | 0.05 | 0.71 | 5 | 1277511 |
| 2009 | 0.54 | 0.14 | 0.93 | 7 | 1261588 |
| 2010 | 0.23 | 0.00 | 0.49 | 3 | 1244870 |
| 2011 | 0.16 | 0.00 | 0.38 | 2 | 1234761 |
| 2012 | 0.32 | 0.01 | 0.64 | 4 | 1224361 |
| 2013 | 0.33 | 0.01 | 0.65 | 4 | 1219363 |
| 2014 | 0.25 | 0.00 | 0.53 | 3 | 1218844 |
| 2015 | 0.42 | 0.05 | 0.78 | 5 | 1226013 |
| 2016 | 0.74 | 0.26 | 1.22 | 9 | 1246847 |
| 2017 | 0.65 | 0.20 | 1.10 | 8 | 1263740 |
| 2018 | 0.57 | 0.15 | 0.99 | 7 | 1273002 |
| 2019 | 0.49 | 0.10 | 0.88 | 6 | 1278692 |
| 2020 | 0.65 | 0.20 | 1.10 | 8 | 1283060 |
| 2021 | 0.41 | 0.05 | 0.76 | 5 | 1285488 |
