# Supplementary figures and images for: Ongoing Increase in Incidence of Diabetes in Austrian Children and Adolescents (1989–2021): Results from a Nationwide Registry
Source: Pediatr Diabetes. 2023 Aug 18;2023:4616903. doi: 10.1155/2023/4616903 (PMC12017070; doi:10.1155/2023/4616903)

Crude Rate/100,000 yrs

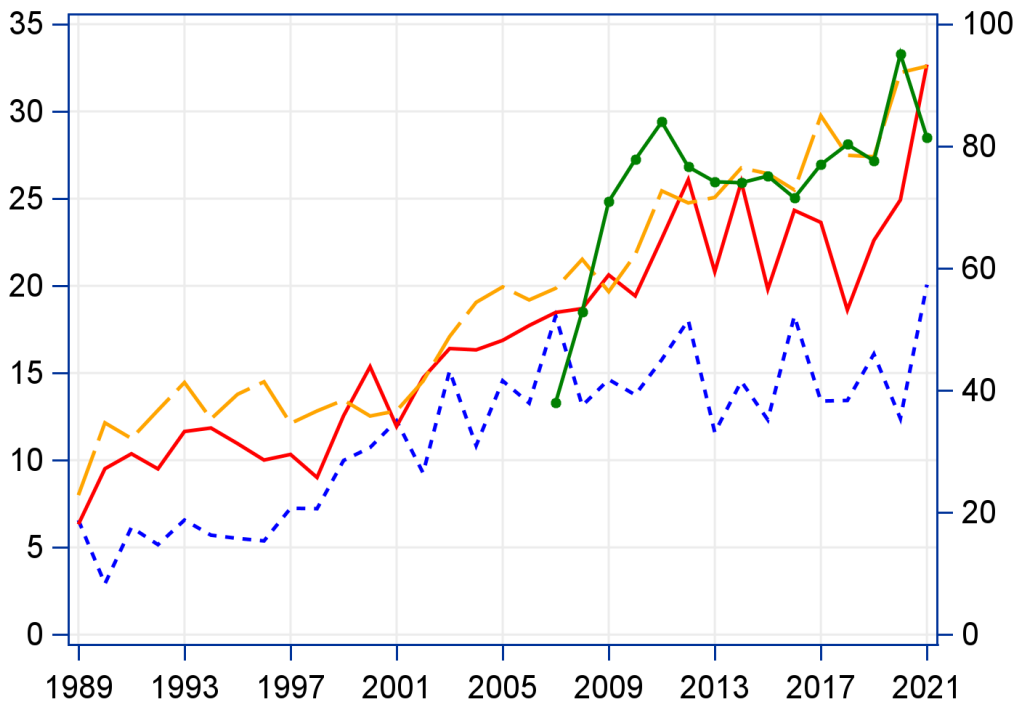

Vaccination coverage %

Year of diagnosis

Age (yrs)

--- 0-4    --- 5-9    --- 10-15    --- Vacc. Rate

Supplement: Supplementary 3 — Crude T1D incidence 1989–2021 and rotavirus vaccination coverage in Austria. [file 4616903.f3.pdf]

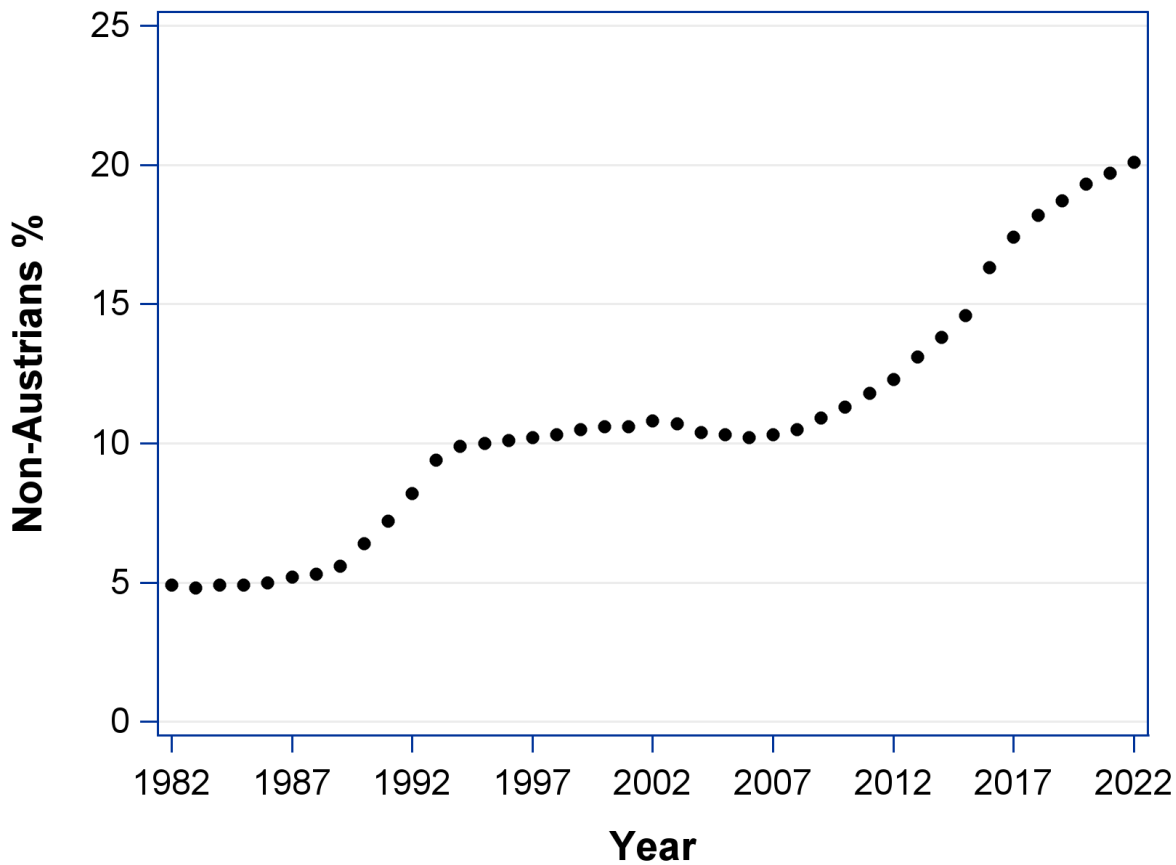

Supplement: Supplementary 4 — Percentage of inhabitants with non-Austrian nationality below 15 years of age in Austria (graphical description). [file 4616903.f4.pdf]
