# Supplementary material for: Ongoing Increase in Incidence of Diabetes in Austrian Children and Adolescents (1989–2021): Results from a Nationwide Registry
Source: Pediatr Diabetes. 2023 Aug 18;2023:4616903. doi: 10.1155/2023/4616903 (PMC12017070; doi:10.1155/2023/4616903)
Supplement: Supplementary 5 — Percentage of inhabitants with non-Austrian nationality below 15 years of age in Austria (tabular format). [file 4616903.f5.docx]

### Suppl. Table 3: Percentage of Inhabitants with Non-Austrian Nationality below 15 years of age in Austria

| Year | % Non-Austrian, aged 0-14 years |
| --- | --- |
| 1982 | 4,9 |
| 1983 | 4,8 |
| 1984 | 4,9 |
| 1985 | 4,9 |
| 1986 | 5,0 |
| 1987 | 5,2 |
| 1988 | 5,3 |
| 1989 | 5,6 |
| 1990 | 6,4 |
| 1991 | 7,2 |
| 1992 | 8,2 |
| 1993 | 9,4 |
| 1994 | 9,9 |
| 1995 | 10,0 |
| 1996 | 10,1 |
| 1997 | 10,2 |
| 1998 | 10,3 |
| 1999 | 10,5 |
| 2000 | 10,6 |
| 2001 | 10,6 |
| 2002 | 10,8 |
| 2003 | 10,7 |
| 2004 | 10,4 |
| 2005 | 10,3 |
| 2006 | 10,2 |
| 2007 | 10,3 |
| 2008 | 10,5 |
| 2009 | 10,9 |
| 2010 | 11,3 |
| 2011 | 11,8 |
| 2012 | 12,3 |
| 2013 | 13,1 |
| 2014 | 13,8 |
| 2015 | 14,6 |
| 2016 | 16,3 |
| 2017 | 17,4 |
| 2018 | 18,2 |
| 2019 | 18,7 |
| 2020 | 19,3 |
| 2021 | 19,7 |
| 2022 | 20,1 |
